# Overexpression of Aiolos promotes epithelial-mesenchymal transition and cancer stem cell-like properties in lung cancer cells

**DOI:** 10.1038/s41598-019-39545-z

**Published:** 2019-02-28

**Authors:** Jung-Jyh Hung, Ying-Shiun Kao, Chi-Hung Huang, Wen-Hu Hsu

**Affiliations:** 1 Division of Thoracic Surgery, Department of Surgery, Taipei Veterans General Hospital and School of Medicine, National Yang-Ming University, Taipei, Taiwan; 2 Taiwan Advance Biopharm (TABP), Inc., Xizhi District, New Taipei City, Taiwan

**Keywords:** Non-small-cell lung cancer, Epithelial-mesenchymal transition

## Abstract

Aiolos/Ikaros family zinc finger 3 (IKZF3), a member of the Ikaros family of lymphocyte maturation-driving transcription factors, is highly expressed in hematopoietic malignancies. However, its role in epithelial-mesenchymal transition (EMT) and cancer stem cell (CSC)-like properties in lung cancer remains unknown. Human lung cancer cell lines H1299 with overexpressing Aiolos (H1299-Aiolos) and A549 with overexpressing Aiolos (A549-Aiolos) were generated by stable transfection. Cell migration and invasion assays were done to demonstrate their invasion and migration ability. Sphere formation assay was used to determine their tumor-initiating capability. Aiolos overexpression induced EMT and increased migration/invasiveness in H1299 and A549 cells. Aiolos overexpression also increased metastatic ability *in vivo*. Aiolos overexpression upregulated the expression of Twist and matrix metalloproteinase 16 (MMP16). By using knockdown of Twist or an inhibitor of phosphatidylinositol (PI) 3-kinase, EMT, migration/invasiveness ability, and MMP16 expression were reversed in H1299-Aiolos and A549-Aiolos cells. Overexpression of Aiolos upregulated the CSC-like properties in lung cancer cells, and were also reversed by an inhibitor of PI 3-kinase. For lung cancer cells, Aiolos overexpression promotes EMT and CSC-like properties through upregulating the PI 3-kinase/Akt pathway. The information is helpful for developing therapeutic strategies targeting Aiolos expression for lung cancer treatment.

## Introduction

Lung cancer is the main cause of cancer-related death worldwide^1^. Surgical resection is the treatment of choice for early-stage non-small cell lung cancer (NSCLC)^2^. Tumor recurrence after surgical resection is the most common cause of treatment failure^3,4^. Epithelial-mesenchymal transition (EMT) is one of the major molecular mechanisms inducing tumor invasion and metastasis^5,6^. Many EMT regulators including Snail, Twist, Slug, Zeb1, SIP1, and E47 were shown to induce EMT through the repression of E-cadherin expression^7–10^. Increased expression of Snail or Twist was associated with tumor recurrence, metastasis and poor prognosis in different types of human cancers^10–15^. The cancer stem cells (CSCs) possess the ability to self-renew and generate secondary tumors, which is described as “tumor-initiating ability”^16^. Recent evidences suggest that the process of EMT generates cells with stem-like properties^17–19^.

The Ikaros family of DNA binding proteins are zinc finger transcription factors playing a critical role in the development and differentiation of specific lineages of hematopoietic cells^20–22^. The involvement of Ikaros family in cancer progression was initially identified in hematopoietic malignancies. Aiolos/Ikaros family zinc finger 3 (IKZF3), a member of the Ikaros family, plays an important role in maturation of B and T cells^23,24^. Elevated Aiolos expression has been reported to promotes cell survival by regulation of Bcl2 family proteins in chronic lymphocytic leukemia^25–27^. Elevated Aiolos expression is detected in follicular center cell lymphomas^28^. Aiolos collaborates with Blimp-1 to regulate the survival of multiple myeloma cells^29^. However, the role of Ikaros family members in solid tumors have not been well demonstrated in the literature. IKZF1 promotes metastatic ability through upregulating Slug and matrix metalloproteinase 2 (MMP2) in ovarian cancer^30^. Li *et al*.^31^ reported that Aiolos decreases expression of a number of integrin and tight junction genes, disrupts cell-cell and cell-matrix interactions, and promotes anchorage independence by silencing SHC1 gene in lung cancer cells^31^. Aiolos overexpression was a prognostic factor of worse survival in patients with NSCLC^31^. However, whether Aiolos expression promotes EMT and CSC-like properties in lung cancer remains unknown.

Since the relationship between Aiolos expression and EMT or CSC-like properties in lung cancer cells has not been well demonstrated, the current study aims to demonstrate the regulating mechanisms Aiolos expression promoting EMT and CSC-like properties in lung cancer cells.

## Results

### Overexpression of Aiolos promotes EMT and metastatic ability in H1299 and A549 cells

**Figure 1:**
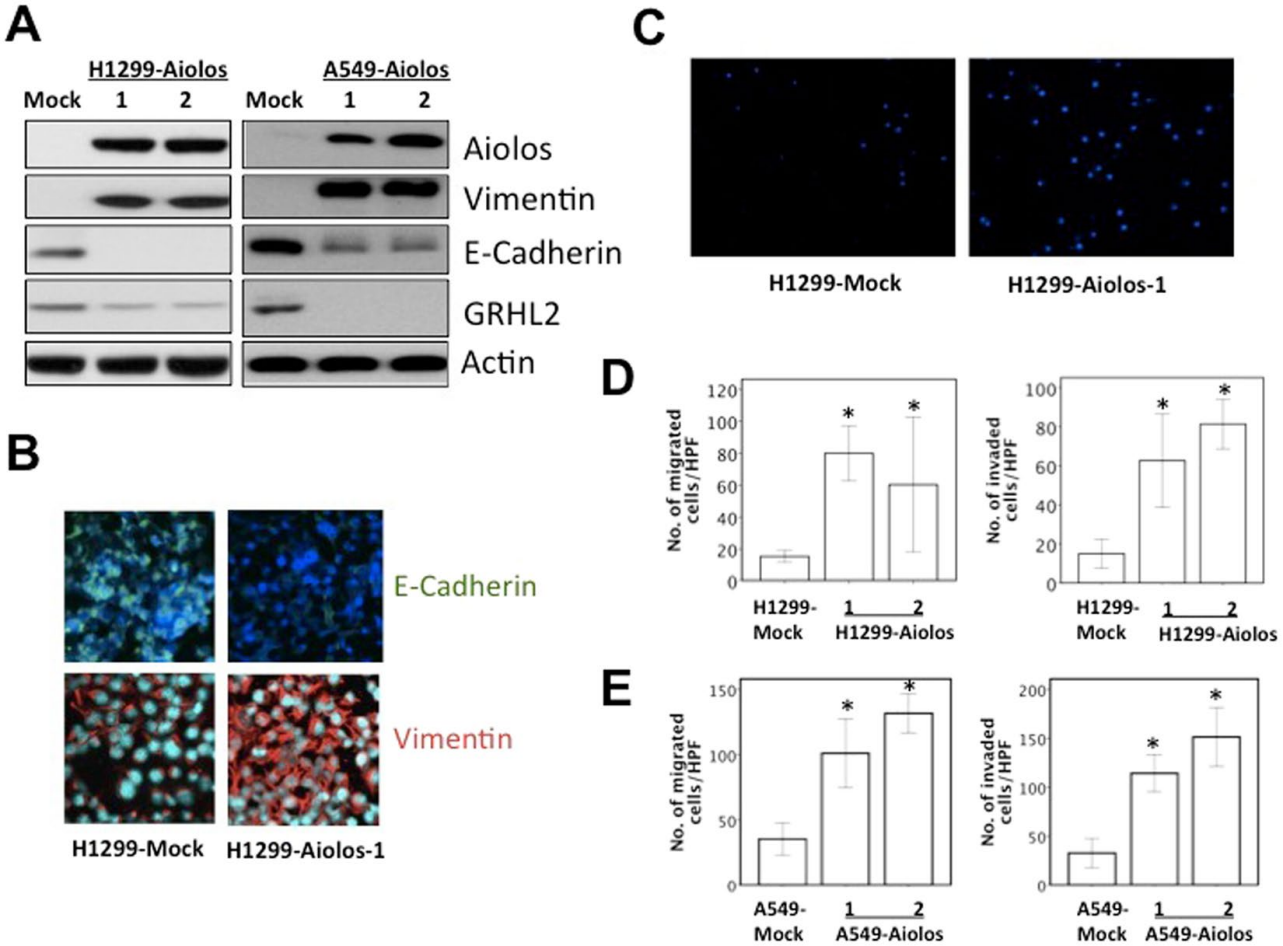
Aiolos overexpression induces EMT and increases migration/invasiveness of H1299 and A549 cells. (**A**) Western blot analysis of Aiolos, Vimentin, E-cadherin, and grainyhead-like 2 (GRHL2) expression in H1299-Aiolos vs H1299-Mock cells and in A549-Aiolos vs A549-Mock cells. Actin was used as a loading control. (**B**) Immuofluorescence staining of E-cadherin and vimentin in H1299-Aiolos vs H1299-Mock cells. The green and red signals represented the staining of E-cadherin and vimentin, respectively. The blue signal represented nuclear DNA staining by Hoechst 33342. (**C**) A representative picture of H1299-Aiolos and H1299-Mock cells that migrated across the Transwell membrane to the other side of the filter. The nuclei of migrated cells were stained with Hoechst 33342. (**D**) The number of H1299-Aiolos vs H1299-Mock cells that migrated across the membrane or invaded across the matrigel counted per high power field. Asterisk indicates *P* < 0.05, compared with control cells (Student’s t-test). (**E**) The number of A549-Aiolos vs A549-Mock cells that migrated across the membrane or invaded across the matrigel counted per high power field. Asterisk indicates *P* < 0.05, compared with control cells (Student’s t-test).

**Figure 2:**
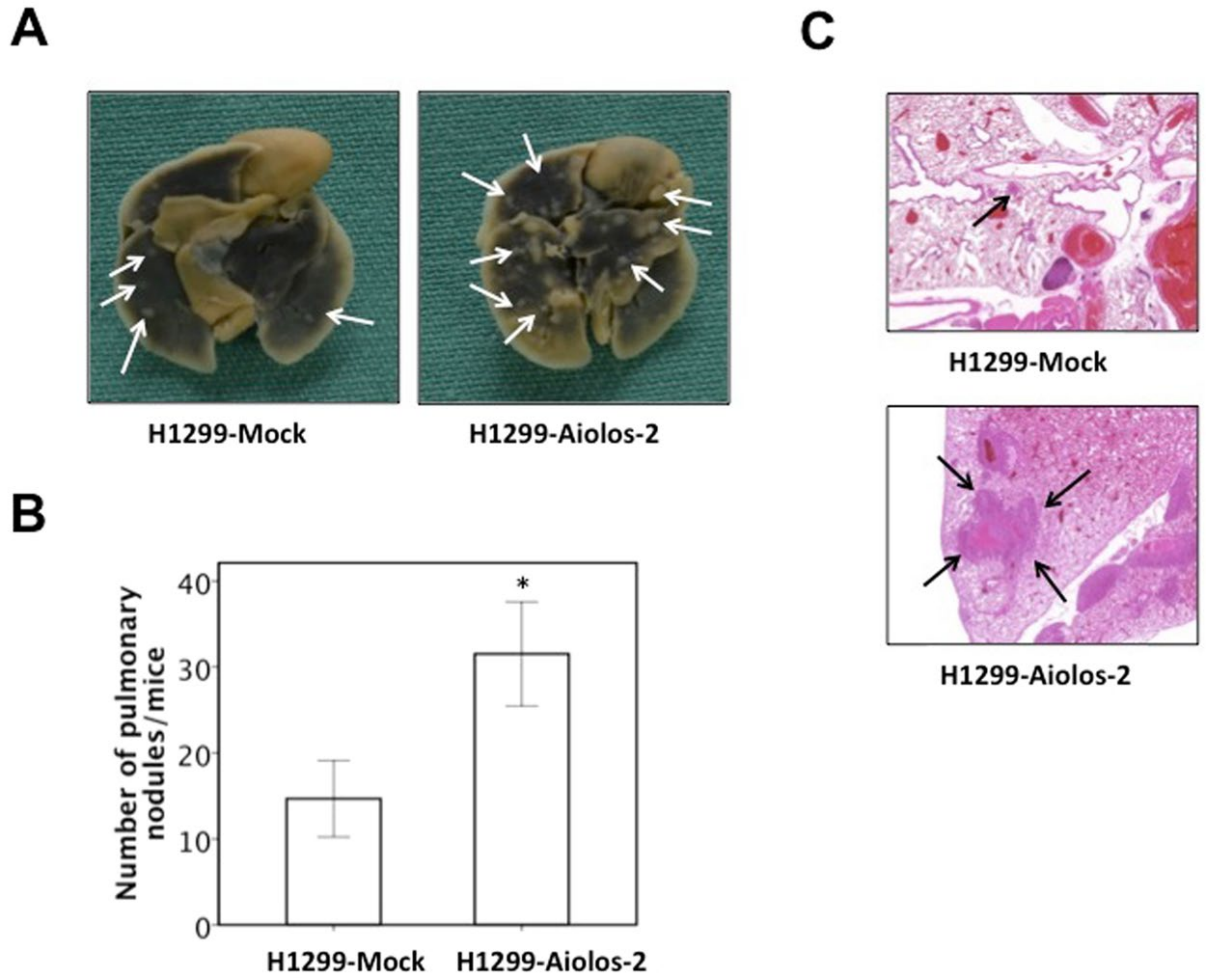
Aiolos overexpression increases metastasis *in vivo*. (**A**) Photographic pictures of the lungs of NOD-SCID mice 16 weeks after tail vein injection of H1299-Mock or H1299-Aiolos cells. White arrows indicated the metastatic nodules. (**B**) Quantification of the average numbers of metastatic foci in the lungs of mice. The asterisk (*) indicated statistical significance (*P* < 0.05) between experimental and control clones. (**C**) H&E staining of lung tissues of mice. Black arrows indicated the metastatic nodules. The photographs were taken at the magnification of x40.

The Aiolos expression was relatively low in lung cancer cell lines in our lab, including H1299, A549, H292, H441, and H520 (Supplementary Fig. 1); therefore, we performed overexpression experiments instead of knockdown experiments. The Aiolos gene was transfected into H1299 and A549 cells, and we established cell lines stably expressing Aiolos (H1299-Aiolos and A549-Aiolos) and mock-transfected cell lines (H1299-Mock and A549-Mock). Increased Aiolos expression was identified in H1299-Aiolos and A549-Aiolos stable clones compared with H1299-Mock and A549-Mock clones by Western blot analysis (Fig. 1A). Overexpression of Aiolos was verified by qRT-PCR (Supplementary Figs 2A and 3A). Western blot analysis showed decreased E-cadherin expression and increased vimentin expression in H1299-Aiolos and A549-Aiolos clones compared with H1299-Mock and A549-Mock cells (Fig. 1A). The decreased E-cadherin and increased vimentin expressions were confirmed by qRT-PCR in H1299-Aiolos (Supplementary Fig. 2B,C) and A549-Aiolos cells (Supplementary Fig. 3B,C). Grainyhead-like 2 (GRHL2), an epithelial-specific transcription factor, is the master programmer of an epithelial phenotype. Our results also showed downregulation of GRHL2 in H1299-Aiolos and A549-Aiolos cells (Fig. 1A). Immunofluorescence staining showed decreased E-cadherin expression in the cell junctions and increased vimentin expression in the cytoplasm in H1299-Aiolos cells (Fig. 1B). We also observed and compared the morphology of H1299, H1299-Aiolos, A549, and A549-Aiolos cells. Both H1299 and A549 cells had relatively preserved cell-cell adhesion and polarity. However, H1299-Aiolos and A549-Aiolos cells showed high percentage of spindle cell-like appearance and more dispersed (data not shown). Then Boyden chamber migration assay was performed to demonstrate whether migration increases in H1299-Aiolos and A549-Aiolos cells. The results revealed that the migration increased in H1299-Aiolos (Fig. 1C,D) and A549-Aiolos cells (Fig. 1E). Matrigel invasion assay was also performed to demonstrate whether invasiveness increases in H1299-Aiolos and A549-Aiolos cells. The results demonstrated that the invasiveness increased in H1299-Aiolos (Fig. 1D) and A549-Aiolos cells (Fig. 1E). We further performed tail vein metastasis assay to demonstrate whether Aiolos overexpression increased metastasis *in vivo*. The mice being injected with H1299-Aiolos cells had significantly more pulmonary nodules than did those with H1299-Mock cells sixteen weeks after injection (Fig. 2A,B). H&E stain has been done to confirm the histology of the pulmonary metastatic nodules (Fig. 2C). All the above results showed that Aiolos overexpression induces EMT and increases metastatic ability in lung cancer cells.

### Aiolos overexpression upregulates Twist/MMP16 expression

**Figure 3:**
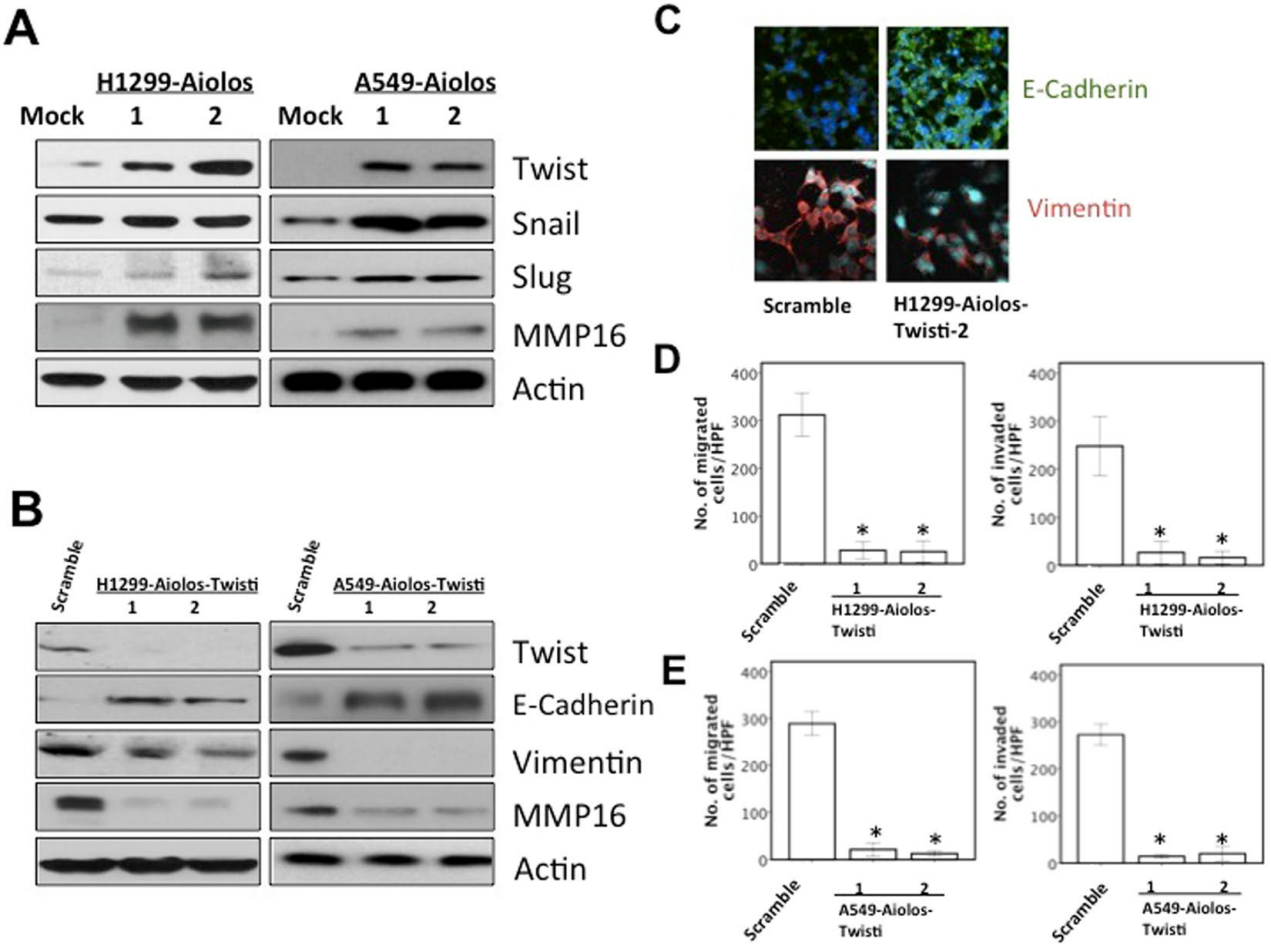
Aiolos overexpression upregulates Twist and MMP16 expression. (**A**) Western blot analysis of Twist, Snail, Slug, and MMP16 expression in H1299-Aiolos vs H1299-Mock cells and in A549-Aiolos vs A549-Mock cells. Actin was used as a loading control. (**B**) Western blot analysis of Aiolos, Twist, E-cadherin, vimentin, and MMP16 in H1299-Aiolos-Scramble and H1299-Aiolos-Twisti clones and in A549-Aiolos-Scramble and A549-Aiolos-Twisti clones. Actin was used as a loading control. (**C**) Immuofluorescence staining of E-cadherin and vimentin in H1299-Aiolos-Scramble vs H1299-Aiolos-Twisti cells. The green and red signals represented the staining of E-cadherin and vimentin, respectively. The blue signal represented nuclear DNA staining by Hoechst 33342. (**D**) The number of H1299-Aiolos-Scrabmle vs H1299-Aiolos-Twisti cells that migrated across the membrane or invaded across the matrigel counted per high power field. Asterisk indicates *P* < 0.05, compared with control cells (Student’s t-test). (**E**) The number of A549-Aiolos-Scrabmle vs A549-Aiolos-Twisti cells that migrated across the membrane or invaded across the matrigel counted per high power field. Asterisk indicates *P* < 0.05, compared with control cells (Student’s t-test).

The protein expression of EMT markers, including Twist, Snail, and Slug, were examined in H1299-Aiolos vs. H1299-Mock cells and in A549-Aiolos vs. A549-Mock cells using Western blot analysis. Twist protein expression was upregulated by Aiolos overexpression (Fig. 3A). Twist mRNA expression was also upregulated by Aiolos overexpression (Supplementary Figs 2D and 3D). We have performed microarray of H1299-Mock and H1299-Aiolos cells (data not shown). MMP16 was among the top uregulated genes in H1299-Aiolos as compared with H1299-Mock cells. We then confirmed the expression of MMP16 in H1299-Aiolos and A549-Aiolos cells by Western blot analysis. The expression of MMP16 was upregulated in H1299-Aiolos and A549-Aiolos cells (Fig. 3A). All these results showed that Aiolos overexpression upregulates the Twist/MMP16 expression, and leads to induction of EMT in lung cancer cells.

### The role of phosphatidylinositol (PI) 3-kinase/Akt/Twist axis in EMT and migration/invasiveness

To further demonstrate the role of Twist expression in EMT phenotypes in H1299-Aiolos and A549-Aiolos cells, we performed knockdown of Twist expression in H1299-Aiolos and A549-Aiolos cells. The result demonstrated that decreased endogenous Twist expression in H1299-Aiolos-Twisti and A549-Aiolos-Twisti cells reversed the EMT phenotype (Fig. 3B). Immunofluorescence staining showed increased E-cadherin expression in the cell junctions and decreased vimentin expression in cytoplasm in H1299-Aiolos-Twisti cells (Fig. 3C). Decreased endogenous Twist expression repressed migration/invasiveness ability in H1299-Aiolos-Twisti and A549-Aiolos-Twisti cells (Fig. 3D,E). Decreased endogenous Twist expression in H1299-Aiolos-Twisti and A549-Aiolos-Twisti cells also suppressed MMP16 expression (Fig. 3B).

**Figure 4:**
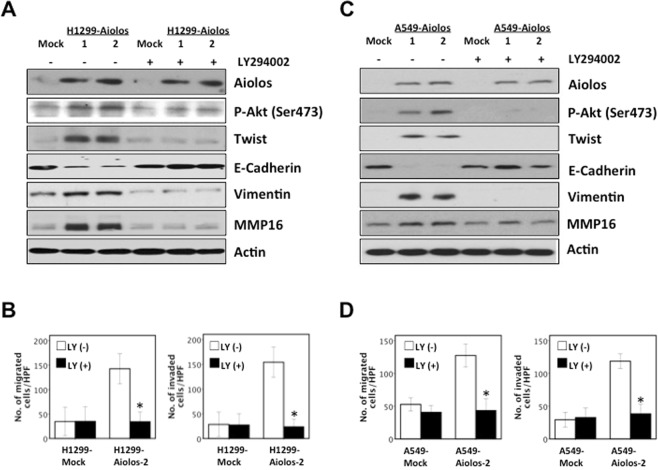
Inhibition of the PI 3-kinase/Akt/Twist axis reverses the EMT phenotype and suppresses migration/invasiveness. (**A**) Western blot analysis of Aiolos, p-Akt, Twist, E-cadherin, vimentin, and MMP16 in H1299-Aiolos vs H1299-Mock clones with and without a PI 3-kinase inhibitor (LY294002) treatment (20 uM for 24 h). (**B**) The number of H1299-Mock vs H1299-Aiolos cells that migrated across the membrane or invaded across the matrigel counted per high power field with and without a PI 3-kinase inhibitor (LY294002) treatment (20 uM for 24 h). Asterisk indicates *P* < 0.05, compared with control cells (Student’s t-test). (**C**) Western blot analysis of Aiolos, p-Akt, Twist, E-cadherin, vimentin, and MMP16 in A549-Aiolos vs A549-Mock clones with and without a PI 3-kinase inhibitor (LY294002) treatment (20 uM for 24 h). (**D**) The number of A549-Mock vs A549-Aiolos cells that migrated across the membrane or invaded across the matrigel counted per high power field with and without a PI 3-kinase inhibitor (LY294002) treatment (20 uM for 24 h). Asterisk indicates *P* < 0.05, compared with control cells (Student’s t-test).

We further examined whether the PI 3-kinase-specific inhibitor, LY294002, blocked the upregulation of Twist/MMP16 in H1299-Aiolos and A549-Aiolos cells. The results demonstrated that inhibition of PI 3-kinase lead to decreased expression of phosphorylated-Akt (Ser473) and Twist, reversion of EMT markers, and decreased expression of MMP16 in H1299-Aiolos cells (Fig. 4A). Inhibition of PI 3-kinase reversed migration/invasion ability increased by Aiolos overexpression in H1299-Aiolos cells (Fig. 4B). In A549-Aiolos cells, inhibition of PI 3-kinase also lead to repression of phosphorylated-Akt (Ser473) and Twist expression, reversion of EMT markers, and decreased expression of MMP16 (Fig. 4C). Inhibition of PI 3-kinase also reversed migration/invasion ability increased by Aiolos overexpression in A549-Aiolos cells (Fig. 4D). All the above results demonstrated that the EMT induced by Aiolos overexpression was regulated through the PI 3-kinase/Akt/Twist axis in lung cancer cells.

To demonstrate the association between Aiolos and Twist expression in human lung adenocarcinoma, immunohistochemical analyses of Aiolos and Twist expression were performed in 93 lung adenocarcinoma samples. Representative immunohistochemical staining of Aiolos and Twist is shown in Supplementary Fig. 4. Expression of Aiolos was shown in 47 (50.5%) of the 93 lung tumor samples. Aiolos expression was significantly associated with Twist expression (*P = *0.005) (Supplementary Fig. 4).

### Overexpression of Aiolos upregulates the CSC-like properties in lung cancer cells

**Figure 5:**
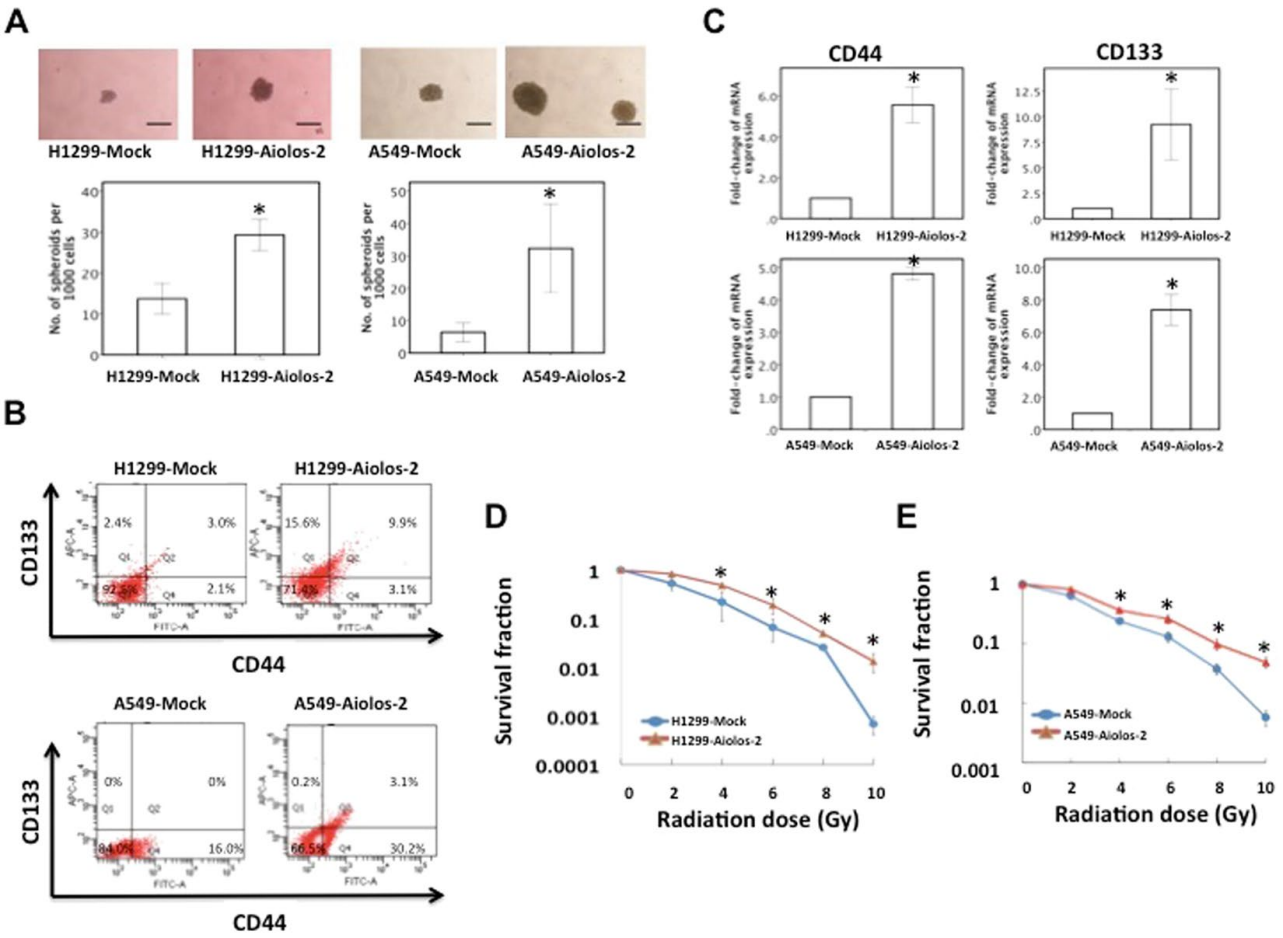
Overexpression of Aiolos upregulates the CSC-like properties in H1299 and A549 cells. (**A**) Representative images (top) and quantification (bottom) of spheroid formation in H1299-Mock and H1299-Aiolos cells and in A549-Mock and A549-Aiolos cells. Scale bars, 100 μm. Asterisk indicates *P* < 0.05, compared with control cells (Student’s t-test). (**B**) Lung cancer stem cell surface markers (CD44 and CD133) in H1299-Aiolos vs H1299-Mock cells and in A549-Aiolos vs A549-Mock cells were analyzed by flow cytometry. (**C**) Real-time PCR analysis of CD44 and CD133 expression in H1299-Aiolos vs H1299-Mock cells (top) and in A549-Aiolos vs A549-Mock cells (bottom). Asterisk indicates *P* < 0.05, compared with control cells (Student’s t-test). (**D**) Survival fraction of H1299-Aiolos cells after irradiation treatment. Asterisks indicate *P* < 0.05, compared with H1299-Mock cells (Student’s t-test). (**E**) Survival fraction of A549-Aiolos cells after irradiation treatment. Asterisks indicate *P* < 0.05, compared with A549-Mock cells (Student’s t-test).

We further investigated the impact of Aiolos on tumor-initiating capability of lung cancer cells. First, we performed sphere formation assay to evaluate the tumor-initiating capability of the cells when Aiolos was overexpressed. In H1299-Aiolos cells, there was a significant increase in formation of spheroids as compared with H1299-Mock cells, which indicates that the cells were capable of initiating tumors when Aiolos was overexpressed (Fig. 5A). There was also a significant increase in formation of spheroids in A549-Aiolos cells as compared with A549-Mock cells (Fig. 5A). Next, we focused to determine the expression of lung cancer CSC surface markers CD44 and CD133 in these lung cancer cells. Both of CD44 and CD133 have been reported to be enriched in lung cancer CSCs. Although we have performed microarray of H1299-Mock and H1299-Aiolos cells, CD44 and CD133 were not available in the microarray data. Therefore, the expression levels of CD44 and CD133 were analyzed by flow cytometry and qRT-PCR. Flow cytometric analysis revealed that ectopic Aiolos expression in H1299 cells increased the CD44^+^ and CD133^+^ populations (Fig. 5B). Fractions of CD44^+^/CD133^+^ cells also increased when Aiolos was overexpressed. Ectopic Aiolos expression in A549 cells also increased the CD44^+^ and CD133^+^ populations (Fig. 5B). qRT-PCR also revealed that the expression levels of CSC surface markers (CD44 and CD133) were significantly increased in H1299-Aiolos cells compared with H1299-Mock cells and in A549-Aiolos cells compared with A549-Mock cells (Fig. 5C). We further investigated the effects of Aiolos overexpression on *in vitro* resistance to irradiation. Clonogenic cell survival assay revealed that the resistance to irradiation was significantly increased when Aiolos was overexpressed in H1299-Aiolos (Fig. 5D) and A549-Aiolos cells (Fig. 5E). We further examined the effect of Aiolos on anchorage-independent proliferation. Aiolos significantly increased anchorage-independent growth in soft agar (Supplementary Fig. 5). Li *et al*.^31^ have shown that Aiolos expression negatively correlated with p66^Shc^ in human lung cancers. Our results also showed that expression of Aiolos in A549 cells repressed p66^Shc^ expression (Supplementary Fig. 5). Similar results were also demonstrated in H1299-Aiolos cells (data not shown).

**Figure 6:**
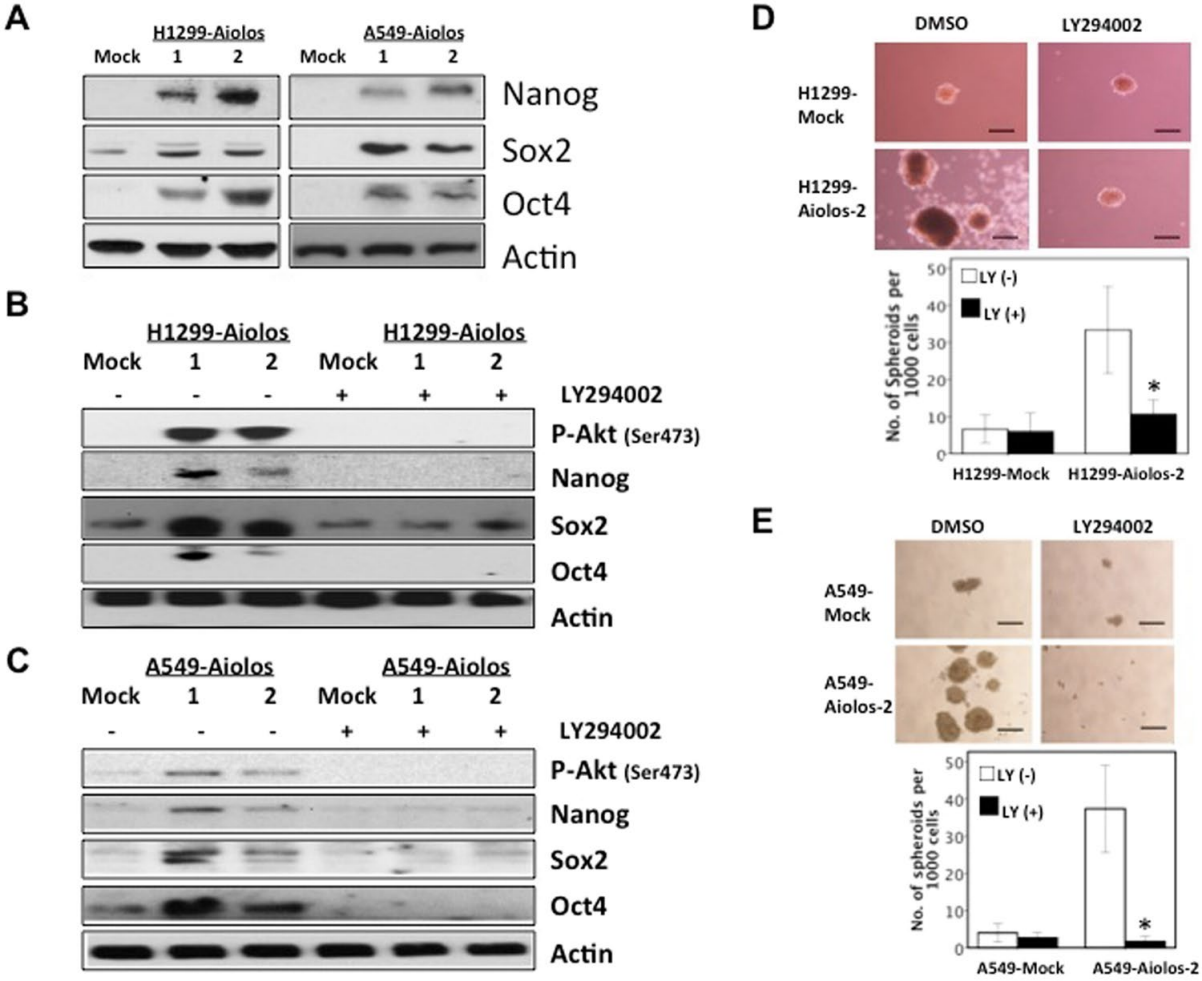
Inhibition of the PI 3-kinase/Akt axis reverses the CSC-like properties. (**A**) Western blot analysis of Nanog, Sox2, and Oct4 expression in H1299-Aiolos vs H1299-Mock cells and in A549-Aiolos vs A549-Mock cells. Actin was used as a loading control. (**B**) Western blot analysis of Nanog, Sox2, and Oct4 in H1299-Aiolos vs H1299-Mock clones with and without a PI 3-kinase inhibitor (LY294002) treatment (20 uM for 24 h). (**C**) Western blot analysis of Nanog, Sox2, and Oct4 in A549-Aiolos vs A549-Mock clones with and without a PI 3-kinase inhibitor (LY294002) treatment (20 uM for 24 h). (**D**) Representative images (top) and quantification (bottom) of spheroid formation in H1299-Mock and H1299-Aiolos cells with and without a PI 3-kinase inhibitor (LY294002) treatment (20 uM). Scale bars, 100 μm. Asterisk indicates *P* < 0.05, compared with control cells (Student’s t-test). (**E**) Representative images (top) and quantification (bottom) of spheroid formation in A549-Mock and A549-Aiolos cells with and without a PI 3-kinase inhibitor (LY294002) treatment (20 uM). Scale bars, 100 μm. Asterisk indicates *P* < 0.05, compared with control cells (Student’s t-test).

Finally, we demonstrated the effects of Aiolos overexpression on the levels of CSC transcription factors, including Oct4, Nanog and Sox2. Western blot analysis showed that Nanog, Oct4, and Sox2 proteins were upregulated in H1299-Aiolos and A549-Aiolos cells (Fig. 6A). Nanog, Oct4, and Sox2 mRNA expressions were also upregulated in A549-Aiolos (Supplementary Fig. 6) and in H1299-Aiolos cells (data not shown). We further examined whether the PI 3-kinase-specific inhibitor, LY294002, repressed the upregulation of Oct4, Nanog, and Sox2 in H1299-Aiolos and A549-Aiolos cells. The results demonstrated that inhibition of PI 3-kinase lead to decreased expression of Oct4, Nanog, and Sox2 proteins in H1299-Aiolos (Fig. 6B) and A549-Aiolos cells (Fig. 6C). Inhibition of PI 3-kinase also reversed the increased number of sphere formation induced by Aiolos overexpression in H1299-Aiolos (Fig. 6D) and A549-Aiolos cells (Fig. 6E). All the above results indicated that Aiolos overexpression promotes the ability of lung cancer cells to develop CSC-like properties, and is regulated through the PI 3-kinase/Akt pathway.

## Discussion

This study showed that overexpression of Aiolos induces EMT, increases migration and invasiveness ability in lung cancer cells through up-regulation of the PI 3-kinase/Akt/Twist axis. Aiolos overexpression also up-regulates CSC-like properties through up-regulating the PI 3-kinase/Akt pathway.

The impact of Aiolos overexpression in hematopoietic malignancies has been reported in the literature^25–29^. In chronic lymphocytic leukemia, Aiolos overexpression has been reported to promotes cell survival by regulation of Bcl2 family proteins^25–27^. Aiolos regulates the survival of multiple myeloma cells by promoting the binding of Blimp-1 to target genes and thereby enhances Blimp-1-dependent transcriptional repression^29^. IKZF1 expression was significantly associated with advanced stage and distant metastasis in ovarian cancer patients^30^. Aiolos overexpression has also been reported to be a poor prognostic factor in patients with NSCLC^31^. Since EMT has been shown to be associated with tumor recurrence, metastasis and poor prognosis in different types of human cancers^10–15^, we focused to demonstrate the regulating mechanisms that Aiolos overexpression promotes EMT and CSC-like properties in the current study.

The relationship between Ikaros family members and EMT has not been well demonstrated in the literature. He *et al*.^30^ have shown that overexpression of IKZF1 significantly upregulated Slug expression and led to increase of migration and invasion in ovarian cancer cells. Li *et al*.^31^ have demonstrated that Aiolos downregulated expression of a number of integrin and tight junction genes and disrupted cell-cell and cell-matrix interactions. In our study, we showed that Aiolos overexpression promoted EMT and metastasis through control of Twist and MMP16. The EMT phenotype could be reversed and the migration/invasiveness ability could be repressed by inhibition of the PI 3-kinase/Akt/Twist axis. Our study was the first in the literature to demonstrate the role of Aiolos in EMT and its regulating mechanisms through control of the PI 3-kinase/Akt/Twist pathway.

The relationship between Aiolos expression and CSC-like properties remains unknown. Li *et al*.^31^ have shown that Aiolos promotes anchorage independence by silencing SHC1 gene in lung cancer cells. In our study, we showed that Aiolos overexpression increased tumor-initiating capability of lung cancer cells. Ectopic Aiolos expression significantly increased CSC surface markers (CD44 and CD133) in lung cancer cells. Furthermore, the resistance to irradiation was significantly increased when Aiolos was overexpressed. Ectopic Aiolos expression also increased Nanog, Oct4, and Sox2 proteins expression. We further showed that the increased CSC-like properties by overexpression of Aiolos were reversed by inhibition of the PI 3-kinase/Akt pathway. Our results demonstrated that increased Aiolos expression promotes the ability of lung cancer cells to develop CSC-like properties through regulation of PI 3-kinase/Akt pathway.

Some limitations of this study should be mentioned. Since Aiolos expression in lung cancer cell lines in our lab was relatively low, we performed overexpression experiments instead of knockdown experiments. Further knockdown or knockout of Aiolos expression in cell lines with Aiolos expression or determine the correlation of EMT characteristics in tumors with Aiolos expression in human lung cancer specimens will be helpful to further determine the regulating mechanisms. Furthermore, subcutaneous inoculation of different numbers of lung cancer cells mixed with matrigel into BALB/c nude mice will help to further confirm the effect of Aiolos on lung cancer cell stemness. In conclusion, we have shown that Aiolos overexpression promotes transformation activity and promotes EMT through control of PI 3-kinase/Akt/Twist axis in lung cancer. Aiolos overexpression also promotes CSC-like properties through control of PI 3-kinase/Akt pathway. The information is helpful for developing therapeutic strategies targeting Aiolos expression for lung cancer treatment.

### Patients and methods

All methods were performed in accordance with the relevant guidelines and regulations. All experimental protocols were approved by Institutional Review Board of Taipei Veterans General Hospital, and the informed consent was waived.

### Cell lines, plasmids, and transfection

The human lung cancer cell lines (H1299 and A549) were obtained from American Type Culture Collection (Manassas, VA, USA). H1299 and A549 cells were cultured in Dulbecco’s minimum essential medium (DMEM) (Corning, Manassas, VA, USA) supplemented with 10% fetal bovine serum (FBS), 100 units/ml penicillin, and 100 **u**g/ml streptomycin. The pcDNA3.1(+)-Aiolos plasmid was generated by insertion of a 1428-bp fragment of the full-length human *Aiolos* cDNA into the HindIII/BamHI sites of pcDNA3.1(+) vector. H1299-Aiolos cell lines were established by transfection of the pcDNA3.1(+)-Aiolos plasmid into H1299 cells, and were selected under G418 (1 mg/ml). A549-Aiolos cell lines were also established by transfection of the pcDNA3.1(+)-Aiolos plasmid into A549 cells, and were selected under G418 (1 mg/ml). Vector control cell lines (H1299-Mock and A549-Mock) were generated by transfecting pcDNA3.1(+) into H1299 and A549 cells. The plasmid pSUPER-Twisti was established by inserting the oligonucleotide of 5′-GATCCCCAGGGCAAGCGCGGCAAGAATTCAAGAGATTCTTGCCGCGCTTGCCCTTTTTTA-3′ into the pSUPER plasmid. By inserting the oligonucleotide of 5′-GATCCCCGTGTCTGTAGGAGTCATCCTTCAAGAGAGGATGACTCCTACAGACACTTTTTA-3′ into the pSUPER plasmid, the plasmid pSUPER-scramble was established. The H1299-Aiolos-Twisti cell lines were established by transfection of the pSUPER-Twisti plasmid into H1299-Aiolos cells, and were selected under puromycin (4 ug/mL). By transfection of the pSUPER-Twisti plasmid into A549-Aiolos cells and being selected under puromycin (4 ug/mL), the A549-Aiolos-Twisti cell lines were also established. The H1299-Aiolos-scramble cell lines were established by transfection of the pSUPER-scramble plasmid into H1299-Aiolos cells. By transfection of the pSUPER-scramble plasmid into A549-Aiolos cells, the A549-Aiolos-scramble cell lines were also established.

### RNA preparation and real-time polymerase chain reaction (PCR)

Total RNA was prepared from the lung cancer cell lines by using TRIzol reagent (Invitrogen, Carlsbad, CA, USA). Reverse transcription (RT) was done using 1 **u**g total RNA isolated from cell lines. Real-time quantitative PCR (qPCR) was performed on the LightCycler 480 Real-Time PCR System (Roche Applied Science, Mannheim, Germany). The primer sequences were as follows: Aiolos, 5′-AGAAGGCCCAGCCAATGAAGATGA-3′ and 5′-TCTCCAACTTAATGTTTT CATATTCA-3′; Vimentin, 5′-CCACCAGGTCCGTGTCCTCGT-3′ and 5′-CGCTGCCCAGGCTGTAGGTG-3′; E-Cadherin, 5′-TTGCACCGGTCGACAA AGGAC-3′ and 5′-TGGAGTCCCAGGCGTAGACCAA-3′; Twist, 5′-AGCTACGCCTTCTCGGTCT-3′ and 5′-CCTTCTCTGGAAACAATGACATC-3′; CD44, 5′-TCCAACACCTCCCAGTATGACA-3′ and 5′-GGCAGG TCTGTGACTGATGTACA-3′; CD133, 5′-CACTACCAAGGACAAGGCGT-3′ and 5′-TCCTTGATCGCTGTTGCCAT-3′; Naong, 5′-AGGTATTTTAGTACTCCAC AAACCA-3′ and 5′-AGTGTCCAGACTGAAATTGAGTAAT-3′; Oct4, 5′-CGCAAGCCCTCATTTCAC-3′ and 5′-CATCACCTCCACCACCTG-3′; Sox2, 5′-CACCCCTGGCATGGCTCTT-3′ and 5′-GAGCTGGCCTCGGACTTGA-3′; GAPDH (glyceraldehyde-3-phosphate dehydrogenase), 5′-ACTCCTCCACCTTT GACGCT-3′ and 5′-ACCCTGTTGCTGTAGCCAAA-3′. The relative expression levels were calculated using the comparative cycle threshold (*C*_*T*_) method (2^*−ΔCT*^).

### Protein extraction and Western blot analysis

For protein extraction, cultured cells were lysed with lysis buffer [50 mM Tris-HCl (pH 7.4), 1% NP-40, 0.25% Na-deoxycholate, 150 mM NaCl, 1 mM EDTA] containing protease inhibitors (GBioscience, Saint Louis, MO, USA). Cell lysates were clarified by centrifugation at 13,000 rpm, 4 °C for 10 minutes. The protein content was determined by Bradford method (Bio-Rad Laboratories, Hercules, CA, USA). For Western blot analysis, 50 ug protein extracts from H1299-Mock, H1299-Aiolos, H1299-Aiolos-Twisti, A549-Mock, A549-Aiolos, and A549-Aiolos-Twisti clones were loaded to 10% SDS-PAGE gels and transferred to nitrocellulose filters. The filters were incubated with an anti-Aiolos antibody (19055-1-AP, Proteintech, Rosemont, IL, USA), an anti-Twist antibody (GTX127310, GeneTex, Irvine, CA, USA), and an anti b-actin antibody (GTX629630, GeneTex, Irvine, CA, USA) as loading control. Other proteins used in the study were listed in Supplementary Table 1. Protein bands were visualized using the enhanced chemiluminescence (ECL) detection system (Pierce Biotech) and exposed to film. All experiments were repeated in triplicate.

### Immunofluorescence

The immunofluorescence was done as described previously^13^. An anti-E-cadherin antibody (#610181, BD Biosciences, Franklin Lakes, NJ, USA) and an anti-vimentin antibody (V6630, Sigma-Aldrich Corp., St Louis, MO, USA) were used in the study (See Supplementary Table 1 for the details of the antibodies). To visualize the location of E-cadherin and vimemtin, Dylight488-conjugated goat anti-mouse immunoglobulin G (IgG) and Dylight594-conjugated goat anti-mouse IgG were used, respectively. Hoechst 33342 (Sigma-Aldrich Corp., St Louis, MO, USA) was used for counterstain of the cell nuclei. A Leica laser scanning confocal microscope was used to capture fluorescence images.

### Migration and invasion assays

Migration and invasion assays were performed as previously described^13^. Boyden chamber of Eight-mm pore size was used. Briefly, cells (2.5 × 10^3^) in 0.5% serumcontaining DMEM were seeded onto the upper chamber. The upper surface of the filter was covered with Matrigel (Corning, Manassas, VA, USA) (1:3 dilution with DMEM) in invasion assay. The cells were allowed to migrate for 12 h and invade for 24 h. After incubation time, cells remained attached to the lower part of the membrane were fixed in 4% formaldehyde, stained with Hoechst 33342 dye (Sigma-Aldrich Corp., St Louis, MO, USA), and counted in 10 random fields under a light microscope at high magnification. Experiments were repeated at least in triplicate.

### ***In vivo*** tail vein metastasis assay

Female non-obese diabetic severe-combined immunodeficiency (NOD-SCID) mice (six weeks of age) were used. The NOD-SCID mice were injected with H1299-Mock vs H1299-Aiolos cells (4 × 10^6^, suspended in 0.1 ml PBS) into the tail vein. There were 6 mice in both groups. The mice were sacrificed after sixteen weeks, and the metastatic lesions in the lungs were examined. The lung tissues were fixed in formalin, embedded in paraffin, and stained with hematoxylin and eosin. With both gross and microscopic examination, the number of pulmonary metastatic lesions in each mouse was counted.

### Immunohistochemistry

Ninety-three patients undergoing surgical resection for lung adenocarcinoma were enrolled in this study. The specimen processing and immunohistochemistry procedures were performed as previously described^32^. For Aiolos, a rabbit polyclonal antibody against Aiolos (19055-1-AP, Proteintech, Rosemont, IL, USA) was used at the dilution of 1:30 and incubated at room temperature for 1 hour. For Twist, a rabbit polyclonal antibody against Twist (GTX127310, GeneTex, Irvine, CA, USA) was used at the dilution of 1:40 and incubated at room temperature for 1 hour. The detection was processed in the Discovery XT automated IHC/ISH slide staining system (Ventana Medical System, Inc. Tucson), by using the ultraView Universal DAB Detection Kit (Ventana Medical System, Inc. Tucson), according to the manufacturer’s instruction. The immunoreactivity of Aiolos and Twist was graded from 0 to 2+ (0, no staining; 1+ , weak staining; 2+ , strong staining) according to nuclear expression and only 2+ was considered as a Aiolos or Twist expression immunohistochemistry result.

### Sphere formation assay

Cell suspensions were plated on ultra-low adherent 6 well plates (Corning, Manassas, VA, USA) at 3 × 10^3^ cells per well in 3 mL medium (DMEM supplemented with 5 mM HEPES, 0.1% sodium bicarbonate, and 0.4% BSA). After 14 days, the spheres were counted under a light microscope at high magnification. The assays were independently repeated at least three times.

### Flow cytometric analysis

To analyze CD44 and CD133 expression, cells were resuspended and incubated with fluorescein isothiocyanate (FITC)-conjugated anti-human/mouse CD44 antibody (11–0441, eBioscience, San Diego, USA) and allophycocyanin-conjugated anti-human CD133 antibody (17–1338, eBioscience, San Diego, USA), respectively. The labeled cells were analyzed using a FACSCalibur flow cytometer (BD Biosciences, New Jersey, USA).

### Radiation treatment and clonogenic cell survival assay

Cells were trypsinized and plated on dishes 16 h before irradiation. The Caesium radiation was delivered by a Model 143–68 137Cs irradiator (JL Shepherd and Associates, San Fernando, CA, USA) at a dose rate of 4 Gy min^−1^. Colonies were stained with crystal violet and counted 14 days after irradiation. A colony was defined as having >50 cells. The surviving fraction was calculated by dividing the number of colonies formed by the number of cells plated, multiplied by plating efficiency.

### Soft agar clonogenicity assay

Anchorage-independent growth of H1299-Mock, H1299-Aiolos, A549-Mock, and A549-Aiolos cells was examined by survival of colonies on soft agar as described previously^33^. Cell numbers of 750 were used. The dishes were incubated for 14 days and colonies were counted.

### Statistical analysis

The independent Student’s t-test was used for comparison of the continuous variables between two groups, and the χ^2^ test was applied for comparison of dichotomous variables. Statistical significance was defined as *P* < 0.05.

## Supplementary information

Supplementary tables and figures

## Data Availability

All materials and data in this manuscript are available to Editorial Board Members and referees.
