# Author Correction: Overexpression of Aiolos promotes epithelial-mesenchymal transition and cancer stem cell-like properties in lung cancer cells

**DOI:** 10.1038/s41598-020-57957-0

**Published:** 2020-01-23

**Authors:** Jung-Jyh Hung, Ying-Shiun Kao, Chi-Hung Huang, Wen-Hu Hsu

**Affiliations:** 1 Division of Thoracic Surgery, Department of Surgery, Taipei Veterans General Hospital and School of Medicine, National Yang-Ming University, Taipei, Taiwan; 2 Taiwan Advance Biopharm (TABP), Inc., Xizhi District, New Taipei City, Taiwan

Correction to: *Scientific Reports* 10.1038/s41598-019-39545-z, published online 28 February 2019

In the original version of this Article, Drs Hung and Kao were incorrectly listed as equally contributing authors. This has now been corrected in the original HTML and PDF versions of the paper, and in the accompanying Supplementary Information.

Additionally, the Acknowledgements section in the original version of this Article was incomplete.

“The authors are grateful to Division of Experimental Surgery, Department of Surgery, Taipei Veterans General Hospital, Taipei, Taiwan, for technical assistance. We would like to thank Biobank of NHRI for providing the tissue samples and related clinical data (all are anonymous) for our research. NHRI Biobank is supported by grants from Ministry of Science and Technology and National Health Research Institutes, Taiwan. The authors are also grateful to Dr. Kou-Juey Wu of China Medical University, Taichung, Taiwan, Dr. Shiu-Feng Huang of NHRI, Miaoli, Taiwan, and Dr. Wen-Juei Jeng of Chang-Gung Memorial Hospital, Taoyuan, Taiwan for their contribution to this article.”

now reads:

“The authors are grateful to Division of Experimental Surgery, Department of Surgery, Taipei Veterans General Hospital, Taipei, Taiwan, for technical assistance. We would like to thank Biobank of NHRI for providing the tissue samples and related clinical data (all are anonymous) for our research. NHRI Biobank is supported by grants from Ministry of Science and Technology and National Health Research Institutes, Taiwan. The authors are also grateful to Dr. Kou-Juey Wu of China Medical University, Taichung, Taiwan, Dr. Shiu-Feng Huang of NHRI, Miaoli, Taiwan, and Dr. Wen-Juei Jeng of Chang-Gung Memorial Hospital, Taoyuan, Taiwan for their contribution to this article.

This work was supported in part by Ministry of Science and Technology (MOST 107-2314-B-010-062; J-.J.H.), the Higher Education Sprout Project by the Ministry of Education (MOE) in Taiwan (107AC-D970; J-.J.H.), the National Yang-Ming University-Far Eastern Memorial Hospital Joint Research Program (107DN10 and 108DN14; J-.J.H), Taipei Veterans General Hospital (V107C-149 and V108C-199; J-.J.H.), the Taipei Veterans General Hospital-National Yang-Ming University-Excellent Physician Scientists Cultivation Program (107-V-B-079; J-.J.H.), Yen Tjing Ling Medical Foundation (CI-106-10 and CI-107-15; J-.J.H.), Li-Yang Sheen Medical Education Memorial Foundation (J-.J.H. and W-.H.H.)."

