# Supplementary material for: Overexpression of Aiolos promotes epithelial-mesenchymal transition and cancer stem cell-like properties in lung cancer cells
Source: Sci Rep. 2019 Feb 28;9:2991. doi: 10.1038/s41598-019-39545-z (PMC6395617; doi:10.1038/s41598-019-39545-z)
Supplement: Supplementary file 1 — Supplementary tables and figures [file 41598_2019_39545_MOESM1_ESM.doc]

**Overexpression of Aiolos promotes epithelial-mesenchymal transition and cancer stem cell-like properties in lung cancer cells**

Jung-Jyh Hung,^1^ Ying-Shiun Kao,^1^ **Chi-Hung Huang,^2^ and Wen-Hu Hsu^1^**

^1^Division of Thoracic Surgery, Department of Surgery, Taipei Veterans General Hospital and School of Medicine, National Yang-Ming University, Taipei, Taiwan

^2^Taiwan Advance Biopharm (TABP), Inc., Xizhi District, New Taipei City, Taiwan.

**Address correspondence to:**

Dr. Jung-Jyh Hung (E-mail: bradley.hung@gmail.com)

Division of Thoracic Surgery, Department of Surgery, Taipei Veterans General Hospital and School of Medicine, National Yang-Ming University, No. 201, Shih-Pai Road, Section 2, Taipei 112, Taiwan

Phone: 886-(2)-2875-7546; Fax: 886-(2)-2873-1488

**Running title:** Aiolos overexpression promotes EMT in lung cancer


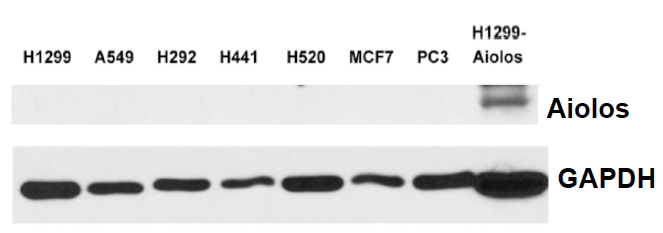
Supplementary Figure 1. Western blot analysis of Aiolos in H1299, A549, H292, H441, H520, MCF7, PC3 and H1299-Aiolos cells. GAPDH was used as a loading control.


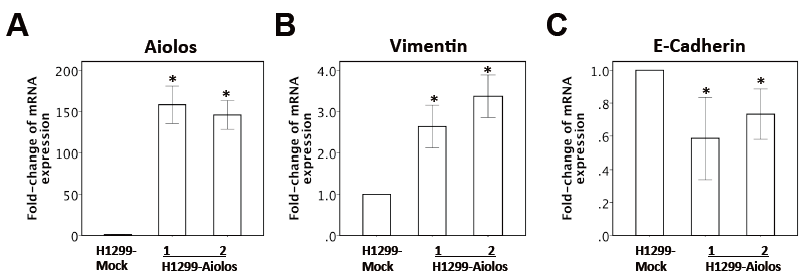


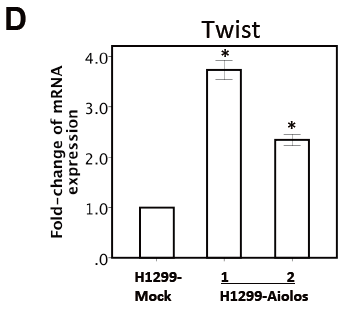


Supplementary Figure 2. Real-time PCR analysis of Aiolos, vimentin, E-Cadherin, and Twist expression in H1299-Aiolos versus H1299-Mock cells. Asterisk indicates *P* <0.001, compared with control cells (Student’s t-test).


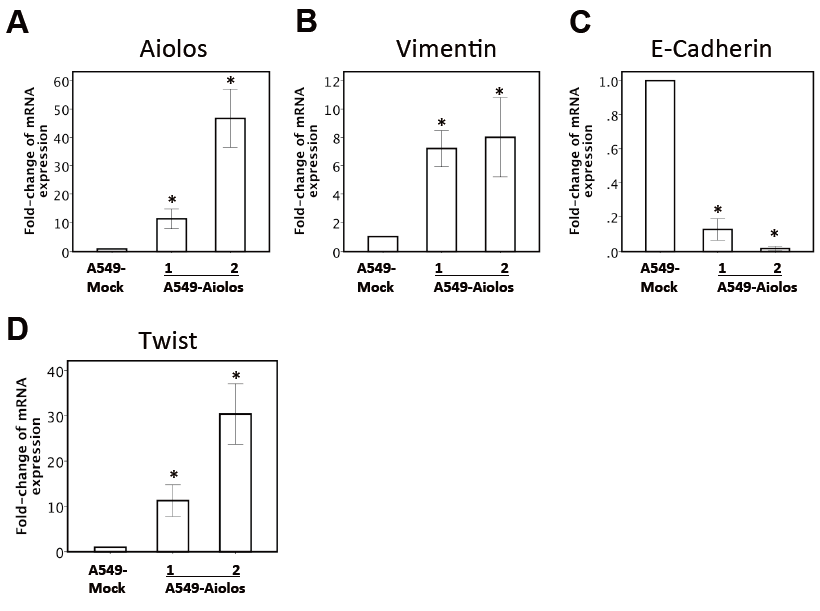
Supplementary Figure 3. Real-time PCR analysis of Aiolos, vimentin, E-Cadherin, and Twist expression in A549-Aiolos versus A549-Mock cells. Asterisk indicates *P* <0.001, compared with control cells (Student’s t-test).


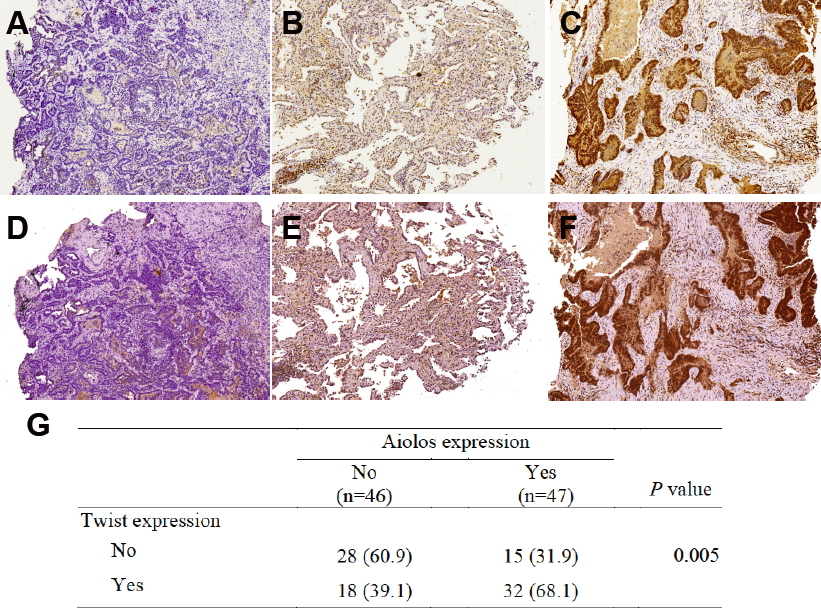
Supplementary Figure 4. Aiolos expression correlated with Twist expression in human lung adenocarcinoma tumors. (A-C) Representative immunohistochemical staining of Aiolos in lung adenocarcinoma tumors scored from (A) 0, (B) 1+, and (C) 2+. Original magnification, x200. (D-F) Representative immunohistochemical staining of Twist in lung adenocarcinoma tumors scored from (D) 0, (E) 1+, and (F) 2+. Original magnification, x200. (G) Correlation between Aiolos expression and Twist expression.


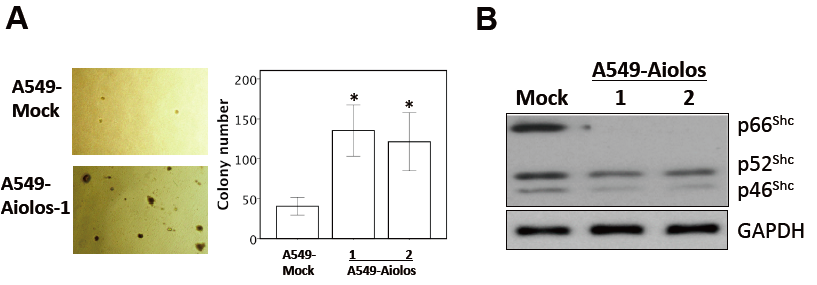
Supplementary Figure 5. Aiolos promotes anchorage independence and negatively correlates with p66^Shc^ expression. (A) A representative picture of soft agar clonogenicity assay (A549-Mock versus A549-Aiolos-1 cells). Asterisk indicates *P* < 0.05, compared with control cells (Student’s t-test). (B) Western blot analysis of p66^Shc^ expression in A549-Aiolos versus A549-Mock cells. GAPDH was used as a loading control.


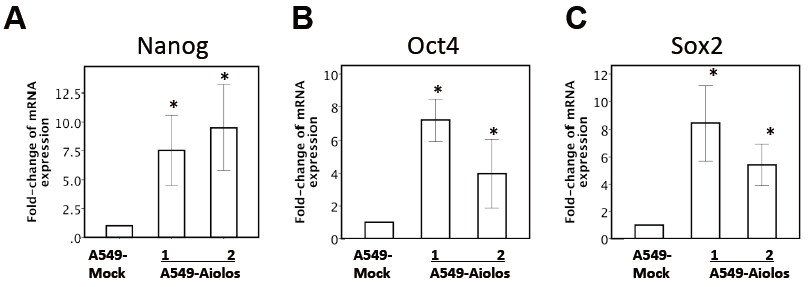
Supplementary Figure 6. Real-time PCR analysis of Nanog, Oct4, and Sox2 expression in A549-Aiolos versus A549-Mock cells. Asterisk indicates *P* <0.001, compared with control cells (Student’s t-test).

Supplementary Table 1. List of proteins tested by antibodies and characteristics of the

corresponding antibodies used

| Protein | Assay | Antibody | Origin | Dilution | Incubation period |
| --- | --- | --- | --- | --- | --- |
| Vimentin | WB | mmab | V6630, Sigma-Aldrich Corp. | 1:500 | overnight |
| Vimentin | IF | rpab | V6630, Sigma-Aldrich Corp. | 1:100 | overnight |
| E-Cadherin | WB | mmab | #610181, BD Biosciences | 1:200 | overnight |
| E-Cadherin | IF | mmab | #610181, BD Biosciences | 1:100 | overnight |
| GRHL2 | WB | rpab | GTX109410, GeneTex, Inc. | 1:1000 | overnight |
| Aiolos | WB | rpab | 19055-1-AP, Proteintech | 1:500 | overnight |
| Aiolos | IHC | rpab | 19055-1-AP, Proteintech | 1:30 | 1 hour |
| Twist | WB | rpab | GTX127310, GeneTex, Inc. | 1:1000 | overnight |
| Twist | IHC | rpab | GTX127310, GeneTex, Inc. | 1:40 | 1 hour |
| b-actin | WB | mmab | GTX629630, GeneTex, Inc. | 1:2500 | 2 hours |
| Snail | WB | rpab | PA5-11923, Thermo Fisher Scientific | 1:500 | overnight |
| Slug | WB | rpab | GTX121924, GeneTex, Inc. | 1:250 | overnight |
| MMP16 | WB | rpab | GTX109378, GeneTex, Inc. | 1:1000 | overnight |
| Phospho-Akt (Ser473) | WB | rpab | #9271, Cell Signaling Technology, Inc. | 1:500 | overnight |
| CD44 | FC | mmab | 11-0441, eBioscience | 1:10 | 30 min |
| CD133 | FC | mmab | 17-1338, eBioscience | 1:10 | 30 min |
| SHC | WB | mmab | #610879, BD Biosciences | 1:4000 | overnight |
| Nanog | WB | rpab | ab80892, Abcam, Inc. | 1:200 | overnight |
| Sox2 | WB | rpab | NB110-37235, Novus Biologicals | 1:500 | overnight |
| Oct4 | WB | rpab | GTX101497, GeneTex, Inc. | 1:250 | overnight |

WB, Western blot; IF, immunofluorescence; IHC, immunohistochemistry; FC, flow cytometry; mmab, mouse monoclonal antibody; rpab, rabbit polyclonal antibody.
